# Influência da termoablação com baixa e alta densidade de energia na junção safeno-femoral, utilizando *laser* endovenoso 1470 nm

**DOI:** 10.1590/1677-5449.010916

**Published:** 2017

**Authors:** Walter Junior Boim de Araujo, Fabiano Luiz Erzinger, Filipe Carlos Caron, Carlos Seme Nejm, Jorge Rufino Ribas Timi

**Affiliations:** 1 Universidade Federal do Paraná – UFPR, Departamento de Cirurgia, Curitiba, PR, Brasil.

**Keywords:** varizes, técnicas de ablação, terapia a *laser*

## Abstract

**Contexto:**

Faz-se importante o conhecimento técnico dos ajustes de potência e de densidade de energia linear endovenosa (*linear endovenous energy density*, LEED) adequados para atingir o objetivo final da termoablação endovenosa (*endovenous laser ablation*, EVLA).

**Objetivos:**

Avaliar a influência de diferentes LEEDs em termos de patência e presença de refluxo, bem como determinar a evolução clínica.

**Métodos:**

Foram incluídas 60 veias safenas magnas (VSM). Os pacientes foram randomizados em dois grupos: EVLA com baixa potência (7 W e LEED de 20-40 J/cm) e com alta potência (15 W e LEED de 80-100 J/cm). O acompanhamento com eco-Doppler e escore de severidade clínica venoso (VCSS) foi realizado nos intervalos de 3-5 dias, 30 dias, 180 dias e 1 ano após o procedimento.

**Resultados:**

Dezoito pacientes (29 membros) tratados com 7W de potência e 13 pacientes (23 membros) com 15 W completaram o estudo. Não houve diferença significativa considerando idade, tempo de cirurgia e o uso de analgésicos, lateralidade, gênero e presença de comorbidades. O LEED médio foi de 33,54 J/cm no grupo de 7 W e de 88,66 J/cm no de 15 W. Ambos apresentaram melhora no VCSS, redução significativa dos diâmetros da JSF e ausência de diferença significativa quanto ao aumento do comprimento do coto da VSM e de refluxo após o tratamento.

**Conclusões:**

A utilização de maior densidade de energia mostrou-se mais efetiva em relação à estabilização do comprimento do coto da VSM e do refluxo em 6 meses. Fazem-se necessários estudos com um período de acompanhamento maior para fundamentar essa hipótese.

## INTRODUÇÃO

A insuficiência venosa crônica causada ​​por varizes é uma condição médica comum, com taxas de prevalência de 28-35% em adultos^1^. As varizes de membros inferiores frequentemente causam desconforto, dor, afastamento do trabalho e deterioração da qualidade de vida^2^^,^ 3.

O tratamento endovenoso através da termoablação da veia safena magna (VSM) com *laser* tem sido utilizado ao longo dos últimos anos em muitos centros em todo o mundo, sendo considerado em alguns deles como primeira opção para o tratamento de varizes, principalmente devido à redução de eventos adversos no pós-operatório^4^.

O principal mecanismo de ação do *laser* é provocar uma reação térmica que pode ser regulada ajustando-se vários parâmetros físicos, tais como comprimento de onda, tipo de administração de energia e quantidade de energia por área de superfície [fluência, expressa em Joule por centímetro quadrado (J/cm^2^)], que dependem da potência, do tempo de duração do pulso e da área de superfície. Devido à importância do tema, outros estudos têm discutido quais os níveis de ajustes de potência e de densidade de energia, comprimento de onda e tipos de fibra de *laser* que seriam adequados para atingir o objetivo final da termoablação endovenosa^5^^-^ 9.

A proposta deste estudo é avaliar a influência de diferentes densidades de energia linear endovenosa (*linear endovenous energy density*, LEED) na evolução ecográfica da junção safeno-femoral (JSF), em termos de patência e presença de refluxo, durante o seguimento de 1 ano após a realização de termoablação da VSM com *laser* endovenoso 1470 nm utilizando 7 W ou 15 W de potência; bem como determinar a evolução clínica e as complicações dos pacientes submetidos ao tratamento proposto.

## MÉTODOS

Trata-se de estudo prospectivo randomizado que avaliou 60 VSMs submetidas a termoablação com *laser* na coxa, com período de seguimento de 12 meses.

O projeto foi previamente aprovado pelo Comitê de Ética em Pesquisa do Hospital de Clínicas da Universidade Federal do Paraná (HC/ UFPR), sob o registro CAAE: 07643012.2.0000.0096, e segue as diretrizes do Ministério da Saúde.

Foram selecionados pacientes com doença venosa crônica dos membros e indicação de tratamento cirúrgico de varizes e que se encaixaram nos critérios de inclusão e exclusão.

Os critérios de inclusão foram: pacientes maiores de 18 anos de ambos os sexos, com diagnóstico e indicação de tratamento cirúrgico para varizes de membros inferiores unilateral ou bilateral, pertencentes à classe C2 a C6 da classificação Clínica, Etiologia, Anatomia e Patofisiologia (CEAP), padronizada para avaliação de doença venosa crônica, e que concordaram em participar do estudo assinando o termo de consentimento.

Os critérios de exclusão foram pacientes com história de trombose venosa profunda e/ou superficial, doença arterial periférica concomitante, dificuldade de deambulação, gestação, amamentação e história de tratamento cirúrgico de varizes.

Após a admissão, os pacientes foram randomizados por data de nascimento. Os nascidos em dias pares foram alocados para serem submetidos a termoablação endovenosa (*endovenous laser ablation*, EVLA) com baixa potência – 7 W e densidade de energia linear endovenosa (LEED) de 20-40 J/cm – e aqueles nascidos em dias ímpares foram alocados para serem submetidos a EVLA com alta potência – 15 W e LEED de 80-100 J/cm. Quando o primeiro grupo (independentemente da potência e LEED utilizados) atingiu 30 VSMs, os demais procedimentos foram realizados no outro grupo até também completar 30 VSMs.

O procedimento foi realizado sob raquianestesia, utilizando fibra convencional de 600 micras de diâmetro e aparelho de *Laser Quanta Systems* com comprimento de onda de 1470 nm regulamentado e com registro na Agência Nacional da Vigilância Sanitária (ANVISA) nº 0520090002. Os pacientes foram submetidos a tratamento de ramos varicosos colaterais no intraoperatório (flebectomia).

**Figura 1:**
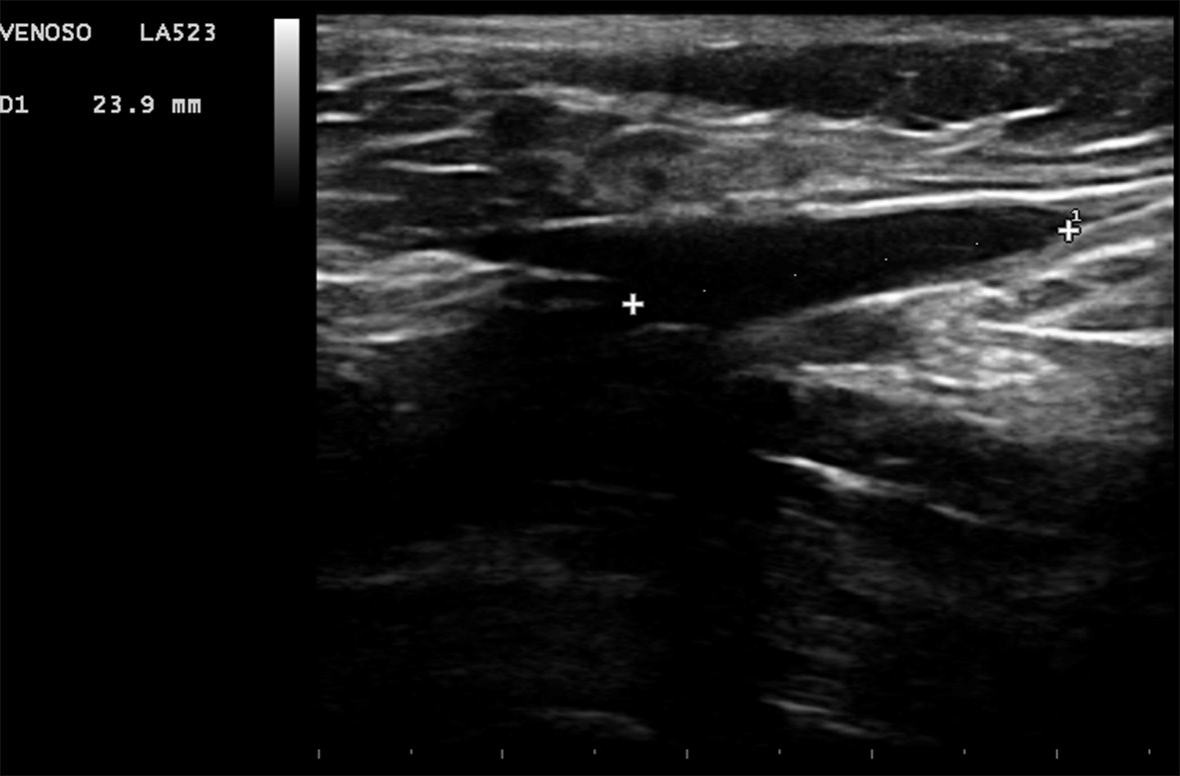
Ponto do início da termoablação visualizado através da hiperecogenicidade da luz a aproximadamente 2 a 2,5 cm da junção safeno-femoral.

Em posição de anti-trendelenburg, a VSM foi cateterizada com agulha de punção (Jelco 16 G). A fibra óptica foi introduzida e conduzida no sentido anterógrado até atingir a região inguinal e a sua extremidade luminosa posicionada próxima ao trígono femoral e sob controle em tempo real do ultrassom foi posicionada entre 2 cm a 2,5 cm da JSF (Figura 1). No caso da não progressão da fibra óptica por impedimento técnico, tortuosidade, dilatação e outros, foram utilizadas técnicas endovasculares com utilização de introdutror, fio guia e cateteres, conforme a necessidade do momento.

**Figura 2:**
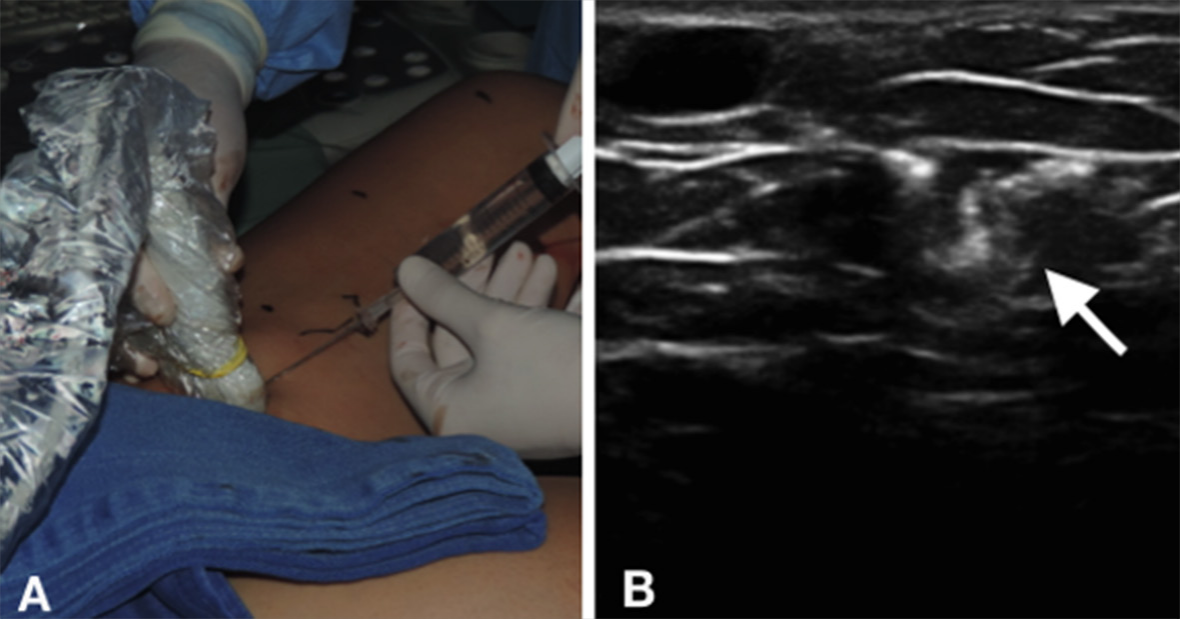
Infiltração com soro fisiológico 0,9% (A) e imagem ecográfica após a infiltração (B); Seta: espaço safênico após tumescência.

Após o posicionamento da fibra, o paciente foi colocado em posição de trendelemburg e foi realizada a tumescência guiada por ultrassom com solução salina fisiológica em temperatura ambiente dentro do espaço safênico (Figura 2).

Sob controle em tempo real por ultrassom, foi realizada a termoablação com aplicação de energia no modo contínuo, com potência previamente determinada (7 ou 15 W). À medida que o ultrassom confirmava a eficácia da termoablação em cada segmento de VSM, a fibra condutora do *laser* era paulatinamente tracionada manualmente no sentido crânio-caudal, em velocidade constante (0,5 mm/seg) e homogênea em todo o trajeto da VSM a ser tratada, sem auxílio de nenhum dispositivo mecânico. Foi registrada a energia total necessária para obtenção da termoablação completa em cada VSM, bem como a extensão de veia tratada, para posterior cálculo da quantidade de energia linear (LEED) utilizada e do tempo gasto para o procedimento.

Após o procedimento, os pacientes foram submetidos a curativo oclusivo, semicompressivo, com malhas e algodão ortopédico, e ataduras de crepe. Os pacientes receberam alta hospitalar no dia seguinte ao do ato cirúrgico, tendo sido prescrito analgésico (ibuprofeno 600 mg de 8 em 8 horas), caso necessário. No segundo dia pós-operatório, os pacientes foram orientados a retirar as faixas e ataduras e colocar meias de média compressão (20-30 mmHg) até a raiz da coxa (7/8) por um período de 60 dias, podendo retirá-las para dormir.

Os pacientes foram reexaminados clinicamente e através de eco-Doppler entre o terceiro e o quinto dia e aos 1, 6 e 12 meses. Todos os pacientes foram submetidos a eco-Doppler de controle pelo mesmo médico ecografista vascular. Apenas o pesquisador estava ciente da potência utilizada em cada paciente. Os critérios clínicos avaliados foram: dor pós-operatória; quantidade de analgesia necessária; tempo de retorno as atividades diárias; satisfação do paciente e presença de eventos adversos (equimose, enduração, trombose venosa profunda, embolia pulmonar, parestesias, queimadura de pele).

Os critérios ecográficos de avaliação da JSF seguiram a proposta de classificação descrita nas diretrizes de prática clínica para o manejo de pacientes com varizes e doenças venosas da *Society for Vascular Surgery* e da *American Venous Forum* (Tabela 1)^10^.

**Tabela 1:** Proposta de classificação dos resultados do eco-Doppler na junção. safeno-femoral após a termoablação.

Patência	J0	Ausência de coto patente
	J1, J2, J3, J4, etc.	Junção com coto patente de 1, 2, 3, 4 cm, etc.
Refluxo	R+	Refluxo
	R-	Sem refluxo

### Análise estatística

Para descrição das variáveis quantitativas, foram consideradas as estatísticas de média, valor mínimo, primeiro quartil, mediana, terceiro quartil, valor máximo e desvio padrão (DP). Para sumarização das variáveis qualitativas, foram consideradas frequências e percentuais. A comparação dos dois grupos, definidos pela potência usada, em relação às variáveis qualitativas foi realizada considerando-se o teste *t* de Student para amostras independentes ou o teste não paramétrico de Mann-Whitney. Já a comparação das potências, em cada momento, quanto à probabilidade de refluxo, foi realizada utilizando o teste exato de Fisher. A avaliação da condição de normalidade das variáveis foi realizada por meio do teste de Jarque-Béra. Valores de p menores do que 0,05 indicaram significância estatística. Os dados foram analisados com o programa computacional IBM SPSS Statistics v.20.

## RESULTADOS

Um total de 31 pacientes conseguiram completar o seguimento de 1 ano do estudo, sendo 18 deles (29 membros) tratados com 7 W de potência e 13 (23 membros) com 15 W. Não houve diferença significativa entre os dois grupos considerando idade, tempo de cirurgia e uso de analgésicos no período de 3-5 dias após a cirurgia. Também não houve diferença quando consideradas as variáveis lateralidade, gênero e presença de comorbidades, mostrando tratar-se de dois grupos homogêneos (Tabela 2).

**Tabela 2:** Resultados da comparação dos dois grupos considerando as variáveis lateralidade, gênero e presença de comorbidades.

	**Potência**		**Valor de p**
	**7 W**	**15 W**	
	**n (%)**	**n (%)**	
Lateralidade			0,353
Bilateral	11 (61,1)	10 (76,9)	
Unilateral	7 (38,9)	3 (23,1)	
Gênero			0,371
Feminino	16 (88,9)	10 (76,9)	
Masculino	2 (11,1)	3 (23,1)	
Comorbidade			0,768
Não	13 (72,2)	10 (76,9)	
Sim	5 (27,8)	3 (23,1)	
Total	18	13	

O LEED médio foi de 33,54 J/cm (DP = 4,5 J/cm; mediana = 33,9 J/cm; mínimo = 23,0 J/cm; máximo = 39,86 J/cm) no grupo de 7 W e de 88,66 J/cm (DP = 12,5 J/cm; mediana = 84,52 J/cm; mínimo = 68,0 J/cm; máximo = 124,0 J/cm) no grupo de 15 W.

**Figura 3:**
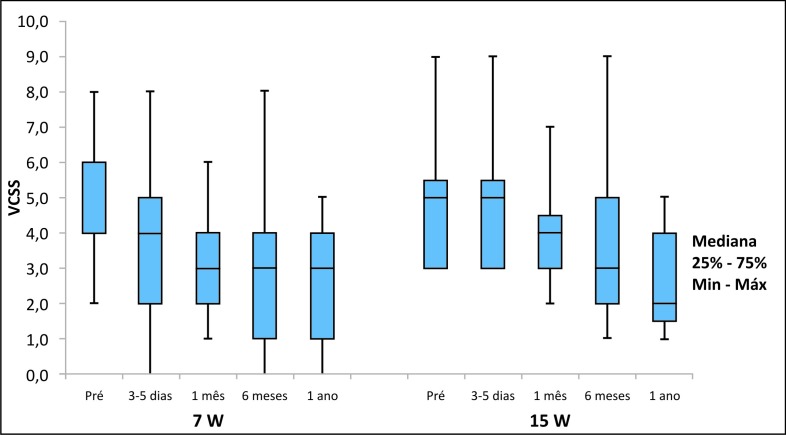
Gráfico com os resultados da comparação dos dois grupos considerando o escore de severidade clinica venosa (VCSS).

No grupo de tratamento com 7 W, a média do escore de severidade clínica venosa (*venous clinical severity score*, VCSS) diminuiu de 4,7 (DP = 2,0) nos valores pré-tratamento para 2,4 (DP = 1,9) após 1 ano do tratamento (p < 0,001). Já no grupo de 15 W, essa média diminuiu de 5,0 (DP = 1,9) para 2,7 (DP = 1,5) após 1 ano (p < 0,001). Não houve diferença significativa entre os grupos quando considerado o VCSS, visto que pacientes dos dois grupos tiveram redução significativa do VCSS (Figura 3).

**Figura 4:**
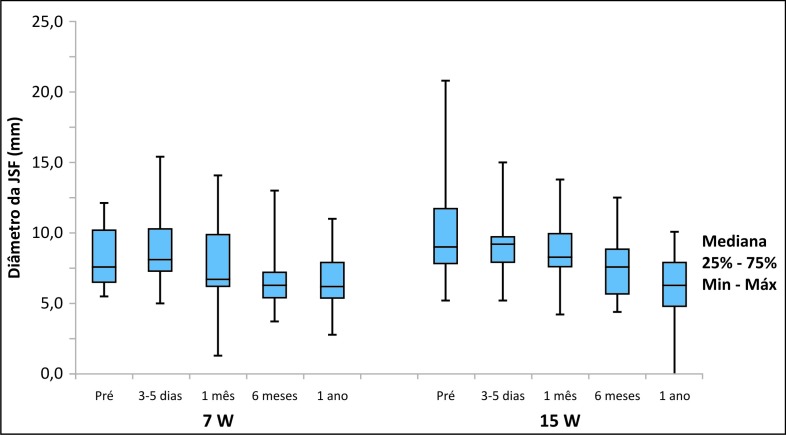
Gráfico com os resultados da comparação dos dois grupos considerando a evolução dos diâmetros da junção safeno-femoral (JSF).

No grupo de tratamento com 7 W, a média dos diâmetros da JSF diminuiu de 8,2 mm (DP = 2,1 mm) nos valores pré-tratamento para 6,6 mm (DP = 2,0 mm) após 1 ano do tratamento (p < 0,001). Já no grupo de 15 W, essa média caiu de 9,9 mm (DP = 3,5 mm) para 5,9 mm (DP = 3,3 mm) após 1 ano (p < 0,001). Portanto, pacientes dos dois grupos tiveram redução significativa dos diâmetros da JSF, e não houve diferença significativa entre os grupos quando considerada a evolução dos diâmetros da JSF (Figura 4).

Pacientes dos dois grupos tiveram aumento significativo do comprimento do coto da VSM ao longo do tempo de acompanhamento. No grupo de tratamento com 7 W, a média aumentou de 1,0 cm (DP = 0,6 cm) para 1,8 cm (55%) (DP = 1,2 cm) após 1 ano do tratamento (p < 0,001). Já no grupo de 15 W, essa média aumentou de 0,7 cm (DP = 0,8 cm) para 1,2 cm (DP = 0,7 cm) após 1 ano (p < 0,048).

**Figura 5:**
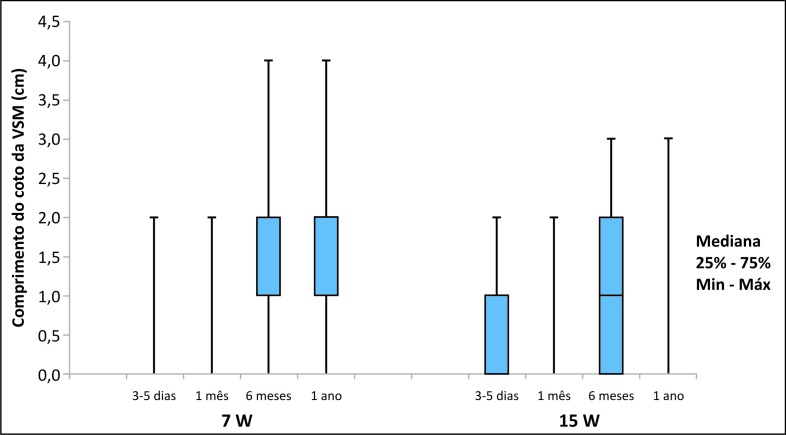
Gráfico com os resultados da comparação dos dois grupos considerando a evolução do comprimento do coto da veia safena magna (VSM) após 1 ano de acompanhamento.

A utilização de maior densidade de energia (LEED médio de 84 J/cm) mostrou-se mais efetiva em relação à estabilização do comprimento do coto da VSM e do refluxo em 6 meses; entretanto, não houve diferença significativa entre os grupos quando considerado 1 ano de acompanhamento (Figura 5).

## DISCUSSÃO

Refluxo na JSF e na VSM é a causa mais comum de varizes primárias, respondendo por 60-70% dos casos^11^. O tratamento cirúrgico convencional de varizes, quando envolve o refluxo na JSF, implica na ligadura e retirada da VSM e de suas tributárias, sendo que ligadura inadequada é sugerida como uma das causas na recorrência de varizes. Portanto, um fator que pode contribuir para a recorrência é a presença de refluxo na veia safena acessória (VSA), devendo nesse caso receber tratamento ablativo, e para alguns autores ser tratada mesmo quando competente^12^.

A fim de alcançar um resultado bem sucedido, durante o tratamento cirúrgico de varizes convencional todas as tributárias da JSF ou proximal da VSM com refluxo demonstraram exigir ligadura. Esse conceito também deveria ser importante quando da realização dos tratamentos endovenosos; pois, se após a termoablação persistir o refluxo da JSF, ele pode ser distribuído para a VSM ou um dos outros afluentes principais, tais como a VSA. No entanto, o refluxo para mais de um afluente importante é incomum, ocorrendo em menos de 5% dos casos, e alguns autores argumentam que a ligadura de tributárias competentes é desnecessária e pode ainda promover neovascularização^13^^,^ 14.

Engelhorn et al.^15^ demostraram que VSM maior que 7 mm está relacionada com refluxo em 71% dos casos e que, quando esta é maior que 9 mm, a probabilidade de refluxo é de 100%. Na termoablação, o grau da lesão da veia safena será induzida pelo calor, que vai depender de dois fatores: da temperatura atingida e do tempo que o aparelho ficará em contato com a parede da veia, ocasionando sua destruição por fototermólise^16^.

Nesse sentido, para uma termoablação mais efetiva possível, principalmente para veias de maiores diâmetros, pode-se fazer necessária a utilização de altas energias, capazes de causar uma redução do diâmetro da safena bem como a sua obliteração definitiva. Pannier et al., em um estudo usando *laser* de 1470 nm com fibra radial, evidenciou uma taxa elevada de oclusão após seis meses, utilizando densidade de energia relativamente alta (LEED de 90,8 J/cm e fluência de 35,5 J/cm^2^)^17^.

Nos pacientes em que utilizamos uma energia maior (LEED médio de 84 J/cm), após 1 ano de seguimento foi evidenciada uma menor incidência de pacientes com refluxo na JSF (17%, quatro pacientes). Já quando foi utilizada uma energia menor (LEED médio de 33 J/cm), o número de pacientes com refluxo na JSF foi maior e também aumentou gradualmente ao longo do tempo (de 1 mês para 1 ano), totalizando nove pacientes. Embora essa diferença não tenha obtido significância estatística, esse fato deixa em aberto a hipótese de que pode ocorrer um aumento progressivo no número de pacientes que venham a apresentar refluxo na JSF nos próximos anos de seguimento, embora isso não possa ser comprovado pelo presente estudo. Achados semelhantes também foram verificados por Proebstle et al.^18^, que demonstraram que o EVLT (*endovenous laser treatment*) com baixa energia foi responsável pelos piores resultados, levando a uma maior taxa de recorrência.

Com relação ao comprimento do coto da VSM ao longo do tempo de acompanhamento, em nosso estudo os pacientes dos dois grupos tiveram aumento significativo desse comprimento. No grupo de tratamento com 7 W, a média aumentou de 1,0 cm (DP = 0,6 cm) para 1,8 cm (DP = 1,2 cm) após 1 ano do tratamento (p < 0,001), e no grupo de 15 W a média aumentou de 0,7 cm (DP = 0,8 cm) para 1,2 cm (DP = 0,7 cm) após 1 ano (p < 0,048). Não houve diferença significativa entre os grupos. No entanto, quando utilizamos baixa densidade de energia, o aumento do comprimento do coto da VSM ocorreu mais tardiamente; já quando foi utilizado alta densidade de energia, o comprimento do coto da VSM estabilizou com 6 meses.

Outro achado deste estudo é que, apesar de existirem diferenças no acompanhamento ecográfico, principalmente quanto a refluxo, o escore de severidade clinica (VCSS) evidenciou melhora com significância estatística em ambos os grupos após 1 ano. Esse achado justifica o uso de maior densidade de energia proximo à JSF e está de acordo com os achados da literatura demonstrando que, ao utilizar *laser* no modo contínuo e um LEED de 80 J/cm na VSM, pode-se evitar a recanalização^19^.

As limitações deste estudo incluem a amostra pequena e heterogênea de pacientes, a utilização de um método de randomização não usual e o tempo de seguimento relativamente curto.

## CONCLUSÃO

Em 1 ano de seguimento, os dois grupos apresentaram melhora significativa do escore de severidade clínica (VCSS), redução significativa dos diâmetros da JSF e ausência de diferença significativa quanto ao aumento do comprimento do coto da VSM até a área de início da termoablação e de refluxo após o tratamento. No entanto, a utilização de maior densidade de energia (LEED médio de 84 J/cm) mostrou-se mais efetiva com relação à estabilização do comprimento do coto da VSM e do refluxo em 6 meses. Fazem-se necessários estudos com um período de acompanhamento maior para fundamentar essa hipótese.
