# Supplementary material for: Influência da termoablação com baixa e alta densidade de energia na junção safeno-femoral, utilizando laser endovenoso 1470 nm
Source: J Vasc Bras. 2017 Jul-Sep;16(3):220–6. [Article in Portuguese] doi: 10.1590/1677-5449.010916 (PMC5868938; doi:10.1590/1677-5449.010916)
Supplement: Translation

# Translation: The influence of low- and high-energy-density intravenous laser ablation on the saphenofemoral junction, with 1470-nm laser

**Authors:** Walter Junior Boim de Araujo, Fabiano Luiz Erzinger, Filipe Carlos Caron, Carlos Seme Nejm, Jorge Rufino Ribas Timi

**Affiliations:** 1 Universidade Federal do Paraná – UFPR, Departamento de Cirurgia, Curitiba, PR, Brazil.

**Keywords:** varicose veins, ablation techniques, laser therapy

## Abstract

**Background:**

It is important to acquire technical knowledge about the power and linear endovenous energy density (LEED) settings needed to achieve the ultimate goal of endovenous laser ablation (EVLA).

**Objectives:**

To evaluate the influence of different LEEDs in terms of patency and presence of reflux and to determine clinical outcomes.

**Methods:**

Sixty great saphenous veins (GSVs) were included. Patients were randomized into 2 groups, low-power EVLA (7 W and LEED of 20-40 J/cm) and high-power EVLA (15 W and LEED of 80-100 J/cm). Patients were followed-up with duplex ultrasound and calculation of venous clinical severity score (VCSS) at 3-5 days, 30 days, 180 days, and 1 year after the procedure.

**Results:**

18 patients (29 limbs) treated with 7 W of laser power and 13 patients (23 limbs) treated with 15 W of laser power completed the study. There was no significant difference regarding age, operating time, use of analgesics, laterality, sex, or presence of comorbidities. Mean LEED was 33.54 J/cm in the 7-W group and 88.66 J/cm in the 15-W group. Both groups exhibited improvements in VCSS and significant reductions in SFJ diameters, and there were no significant difference in increase of length of the GSV stump or rates of reflux after treatment.

**Conclusions:**

The higher energy density setting was more effective for stabilizing the length of the GSV stump and was associated with a lower incidence of reflux at 6 months. Further studies with a longer follow-up period are required to substantiate this hypothesis.

## INTRODUCTION

Chronic venous insufficiency caused by varicose veins is a common medical condition, with prevalence rates of 28-35% in adults.^1^ Varicose veins in the lower limbs often cause discomfort, pain, time off work, and reduced quality of life.^2^^,^ 3

Over recent years, many centers all over the world have adopted endovenous treatment by thermal ablation of the great saphenous vein (GSV) with lasers and many of them consider it the first choice option for treatment of varicose veins, primarily because of the reduced incidence of adverse events during the postoperative period.^4^

The laser’s principal mechanism of action is provocation of a thermal reaction, which can be controlled by adjusting several physical parameters, such as wavelength, type of administration of energy, and quantity of energy per unit of surface area (fluence, expressed in Joules per square centimeter [J/cm^2^]), which are dependent on power, duration of pulse, and surface area. The importance of this subject has prompted many studies to discuss the ideal power settings, energy density, wavelength, and laser fiber types for achieving the final objective of endovenous thermoablation.^5^^-^ 9

The objectives of this study are to evaluate the influence of different linear endovenous energy densities (LEED) on echographic results at the saphenofemoral junction (SFJ) and their evolution, in terms of patency and presence of reflux, over a 1-year follow-up period after thermoablation of the GSV with a 1470 nm endovenous using 7 W or 15 W power settings; and to determine the clinical progress of patients and their complications.

## METHODS

This is a randomized prospective study of 60 GSVs treated with laser thermal ablation in the thigh, with a 12-month follow-up period.

The project was approved in advance by the Research Ethics Committee at the Hospital de Clínicas da Universidade Federal do Paraná (HC/ UFPR), under ruling number CAAE: 07643012.2.0000.0096, and is in compliance with Ministry of Health guidelines.

Patients were selected who had chronic venous disease of the limbs, indications for surgical treatment of varicose veins, and met the inclusion and exclusion criteria.

The inclusion criteria were: patients over the age of 18 years, of both sexes, with diagnoses and indications for surgical treatment of unilateral or bilateral varicose veins of the lower limbs, classified as C2 to C6 on the Clinical, Etiology, Anatomy and Pathophysiology (CEAP) classification, standardized for assessment of chronic venous disease, and who agreed to take part in the study, signing a consent form.

The exclusion criteria were patients with a history of deep and/or superficial venous thrombosis, concomitant peripheral arterial disease, difficulty walking, pregnancy, breastfeeding, and history of surgical treatment of varicose veins.

After admission, patients were randomized by date of birth. Those born on even-numbered dates were allocated to endovenous thermoablation (endovenous laser ablation, EVLA) with a low power setting of 7 W and a linear endovenous energy density (LEED) of 20-40 J/cm. Patients born on odd-numbered dates were allocated to EVLA with a high power setting of 15 W and a LEED of 80-100 J/cm. When one of the groups, irrespective of power and LEED, reached 30 GSVs treated, all subsequent procedures were allocated to the other group until it also numbered 30 GSVs treated.

The procedures were conducted under spinal anesthesia, using a conventional 600 micron diameter fiber and a Laser Quanta Systems unit with a regulated wavelength of 1470 nm, with National Sanitary Surveillance Agency (ANVISA) registration number 0520090002. Patients also underwent treatment of collateral varicose branches intraoperatively (by phlebectomy).

With the patient in the reverse Trendelenburg position, the GSV was catheterized with a puncture needle (Jelco 16 G). The optical fiber was introduced and conducted in the antegrade direction as far as the groin until its light-emitting tip was close to the femoral triangle and, under real-time ultrasound control, it was positioned 2 cm to 2.5 cm from the SFJ (Figure 1). In cases in which the optical fiber could not be advanced because of technical impediments, tortuosity, dilatation, or other reasons, endovascular techniques were employed, with sheaths, guidewires, and catheters, as required in each case.

**Figure 1:** Initial thermal ablation point seen as hyperechogenic lumen approximately 2 to 2.5 cm from the saphenofemoral junction.

Once the fiber was in place, the patient was placed in the Trendelenburg position and ultrasound-guided tumescence was administered with saline solution at room temperature into the saphenous space (Figure 2).

**Figure 2:** Infiltration with 0.9% saline (A) and echographic image after infiltration (B); Arrow: saphenous space after tumescence.

Under real-time ultrasound control, thermal ablation was performed with energy administered in continuous mode at a preset power level (7 or 15 W). As the efficacy of thermal ablation was confirmed by ultrasound for each segment of the GSV, the laser fiber was gradually tractioned manually in the distal direction at a constant velocity (0.5 mm/sec), uniformly along the entire length of the GSV being treated, without assistance from any type of mechanical device. The total quantity of energy needed to achieve total thermal ablation of each GSV was recorded together with the length of vein treated, to enable calculation of the linear energy (LEED) applied, and the time taken to complete the procedure.

After the procedure, patients’ legs were wrapped with occlusive, semicompressive dressings, using orthopedic cotton and gauze, and crepe bandages. Patients were discharged from hospital the day after surgery, with prescriptions for analgesics (600 mg of ibuprofen every 8 hours) in case of need. Two days after the operation patients were instructed to remove the bandages and bindings and wear medium compression stockings (20-30 mmHg) up to the top of the thigh (7/8) for 60 days, removing them to sleep if preferred.

Patients underwent clinical and Doppler ultrasonography examinations between the third and the fifth day after surgery and at 1, 6, and 12 months. All patients underwent control Doppler ultrasonography examinations performed by the same physician, a vascular ultrasound specialist. Only the researcher was aware of the power setting used for each patient. The clinical criteria analyzed were: postoperative pain; quantity of analgesia needed; time taken to return to daily activities; patient satisfaction and occurrence of adverse events (ecchymosis, hardening, deep venous thrombosis, pulmonary embolism, paresthesias, skin burns).

The echographic criteria used for assessment of the SFJ were those in the classification proposed in the clinical practice guidelines for management of patients with varicose veins and venous diseases published by the Society for Vascular Surgery and the American Venous Forum (Table 1).^10^

**Table 1:** Proposal for classification of results of Doppler ultrasonography examination of the saphenofemoral junction after thermal ablation.

Patency	J0	No patent stump
	J1, J2, J3, J4, etc.	Junction with stump patent to 1, 2, 3, 4 cm, etc.
Reflux	R+	Reflux
	R-	No reflux

### Statistical analysis

Quantitative variables were expressed as means, minimum value, first quartile, median, third quartile, maximum values, and standard deviations (SD). Qualitative variables were summarized as frequencies and percentages. The two study groups, defined by the power administered, were compared using Student’s *t* test for independent samples or the Mann-Whitney nonparametric test for qualitative variables. Fisher’s exact test was used to compare the different power levels in terms of the probability of reflux at each point in time. The normality of variables was examined using the Jarque- Béra test. P values below 0.05 indicate statistical significance. Data were analyzed with IBM SPSS Statistics v.20.

## RESULTS

A total of 31 patients managed to complete 1-year follow-up, 18 of whom (29 limbs) had been treated with the 7 W power setting and 13 (23 limbs) with the 15 W setting. There were no significant differences between the two groups for age, duration of surgery, or use of analgesics during the 3-5 day period after surgery. There were also no differences in the variables laterality, sex, or presence of comorbidities, showing that these two groups were homogenous (Table 2).

**Table 2:** Results of comparison of two groups considering the variables laterality, sex, and presence of comorbidities.

	**Power**		**p**
	**7 W**	**15 W**	
	**n (%)**	**n (%)**	
Laterality			0.353
Bilateral	11 (61.1)	10 (76.9)	
Unilateral	7 (38.9)	3 (23.1)	
Sex			0.371
Female	16 (88.9)	10 (76.9)	
Male	2 (11.1)	3 (23.1)	
Comorbidity			0.768
No	13 (72.2)	10 (76.9)	
Yes	5 (27.8)	3 (23.1)	
Total	18	13	

Mean LEED in the 7 W group was 33.54 J/cm (SD = 4.5 J/cm; median = 33.9 J/cm; minimum = 23.0 J/cm; maximum = 39.86 J/cm) and 88.66 J/cm (SD = 12.5 J/cm; median = 84.52 J/cm; minimum = 68.0 J/cm; maximum = 124.0 J/cm) in the 15 W group.

In the group treated with 7 W, the mean venous clinical severity score (VCSS) reduced from a pretreatment baseline of 4.7 (SD = 2.0) to 2.4 (SD = 1.9) 1 year after treatment (p < 0.001). In the 15 W group, mean VCSS fell from 5.0 (SD = 1.9) to 2.7 (SD = 1.5) after 1 year (p < 0.001). There was no significant difference between the groups in terms of VCSS, since patients in both groups had significant reductions in VCSS (Figure 3).

**Figure 3:** Graph showing results of comparison of the groups considering the venous clinical severity score (VCSS).

In the 7 W treatment group, mean SFJ diameter reduced from a pretreatment baseline of 8.2 mm (SD = 2.1 mm) to 6.6 mm (SD = 2.0 mm) 1 year after treatment (p < 0.001). In the 15 W group, this mean fell from 9.9 mm (SD = 3.5 mm) to 5.9 mm (SD = 3.3 mm) 1 year later (p < 0.001). Therefore, patients in both groups exhibited a significant reduction in SFJ diameter, and there was no significant difference between the groups in terms of change in SFJ diameter (Figure 4).

**Figure 4:** Graph showing results of comparison of the groups considering change in diameter of saphenofemoral junction (SFJ).

Patients in both groups exhibited significant increases in the length of the GSV stump over the follow-up period. In the group treated with 7 W, mean length increased from 1.0 cm (SD = 0.6 cm) to 1.8 cm (55%) (SD = 1.2 cm) 1 year after treatment (p < 0.001). In the 15 W group, this mean increased from 0.7 cm (SD = 0.8 cm) to 1.2 cm (SD = 0.7 cm) 1 year later (p < 0.048).

Administration of the greater energy density (a mean LEED of 84 J/cm) proved more effective in relation to stabilization of GSV stump length and to reflux at 6 months; however, there was no significant difference between the groups at 1 year follow-up (Figure 5).

**Figure 5:** Graph showing results of comparison of the groups considering change in length of great saphenous vein (GSV) stump over 1 year follow-up.

## DISCUSSION

Reflux at the SFJ and through the GSV is the most common cause of primary varicose veins, accounting for 60-70% of cases.^11^ When varicose veins involve reflux at the SFJ, conventional surgical treatment requires ligature and removal of the GSV and its tributaries and inadequate ligature has been suggested as one of the causes of recurrence of varicose veins. Therefore, one factor that can contribute to recurrence is reflux in the accessory saphenous vein (ASV), which should be treated with ablation in these cases, and according to some authors should be treated even if competent.^12^

In order to achieve successful results, all SFJ or proximal GSV tributaries in which reflux is demonstrated must be ligated during surgical treatment of conventional varicose veins. This should also be considered important when endovenous treatment is conducted; since if reflux remains at the SFJ after thermal ablation, it could be transferred into the GSV or another of its principle affluents, such as the ASV. However, reflux through more than one important affluent is uncommon, occurring in less than 5% of cases, and some authors argue that ligature of competent tributaries is unnecessary and can provoke neovascularization.^13^^,^ 14

Engelhorn et al. demonstrated that GSVs larger than 7 mm are related to reflux in 71% of cases and that when they are larger than 9 mm, the probability of reflux is 100%.^15^ In thermal ablation, the degree of damage that will be induced by heat in the saphenous vein is dependent on two factors: the temperature reached and the length of time that the fiber is in contact with the wall of the vein, causing destruction by photothermolysis.^16^

Therefore, achieving the most effective thermal ablation possible, particularly in larger diameter veins, may require use of high energies that are capable of causing reduction in the diameter of the saphenous vein and its definitive obliteration. Pannier et al. conducted a study using a 1470 nm laser with a radial fiber, observing a high occlusion rate at 6 months with a relatively high energy density (LEED of 90.8 J/cm and fluence of 35.5 J/cm^2^).^17^

Among the patients to whom we administered the higher energy level (mean LEED of 84 J/cm), at 1 year follow-up there was a smaller incidence of patients with reflux at the SFJ (17%, four patients). Among the patients administered the lower energy level (mean LEED of 33 J/cm), the number of patients with reflux at the SFJ was higher and also gradually increased over time (from 1 month to 1 year), reaching a total of nine patients. Although the difference did not reach statistical significance, this fact raises the hypothesis that there may be a progressive increase in the number of patients who will present with reflux at the SFJ over the coming years of follow-up, although this cannot be confirmed in the present study. Similar findings were also reported by Proebstle et al., who showed that endovenous laser treatment (EVLT) with low energy was responsible for worse results, leading to a higher rate of relapse.^18^

With relation to the length of the GSV stump over the follow-up period, in our study, patients in both groups exhibited significant increases in length. In the group treated with 7 W, mean length increased from 1.0 cm (SD = 0.6 cm) to 1.8 cm (SD = 1.2 cm) 1 year after treatment (p < 0.001) and in the 15 W group, this mean increased from 0.7 cm (SD = 0.8 cm) to 1.2 cm (SD = 0.7 cm) 1 year later (p < 0.048). There was no significant difference between the groups, but in the patients for whom we used lower energy density, the length of the GSV stump increased later, whereas in those administered the higher energy density, the length of the GSV stump stabilized at 6 months.

Another finding in this study is that although differences were seen in follow-up echographic examinations, primarily related to reflux, the clinical severity score (VCSS) revealed statistically significant improvements in both groups after 1 year. This finding justifies use of greater energy density close to the SFJ and is in line with findings in the literature demonstrating that recanalization can be avoided using the laser in continuous mode and a LEED of 80 J/cm in the GSV.^19^

This study’s limitations include the small and heterogeneous sample of patients, the use of an unusual randomization method, and the relatively short follow-up period.

## CONCLUSIONS

Over 1 year follow-up, both groups exhibited significant improvement in the clinical severity score (VCSS), a significant reduction in SFJ diameters, and no significant difference in increased length of the GSV stump up to the initial area of thermal ablation, or reflux after treatment. Notwithstanding, use of a higher energy density (mean LEED of 84 J/cm) proved more effective in terms of stabilization of the length of the GSV stump and of reflux at 6 months. Studies with longer follow-up periods are needed to confirm this hypothesis.
